# Prevalence and distribution of *bla*_CTX-M_, *bla*_SHV_, *bla*_TEM_ genes in extended- spectrum β- lactamase- producing *E. coli* isolates from broiler farms in the Philippines

**DOI:** 10.1186/s12917-019-1975-9

**Published:** 2019-07-05

**Authors:** Romeo S. Gundran, Paul A. Cardenio, Marvin A. Villanueva, Fredelon B. Sison, Carolyn C. Benigno, Kwanchai Kreausukon, Duangporn Pichpol, Veerasak Punyapornwithaya

**Affiliations:** 1 College of Veterinary Science and Medicine, Central Luzon State University, 3120 Science City of Munoz, Nueva Ecija Philippines; 2 Department of Agriculture, Livestock Biotechnology Center, 3120 Science City of Munoz, Nueva Ecija Philippines; 3 Food and Agriculture Organization of the United Nations Regional Office for Asia and the Pacific, Bangkok, Thailand; 4 Faculty of Veterinary Medicine, Chiang Mai University, Chiang Mai, 50200 Thailand; 5 Ph.D. Program in Veterinary Science, Faculty of Veterinary Medicine, Chiang Mai University, Chiang Mai, Thailand

**Keywords:** Broiler, *bla*_CTX-M_, *E. coli*, ESBL, *bla*_SHV_, *bla*_TEM_

## Abstract

**Background:**

Antimicrobial resistance is a worldwide problem causing serious health threats. *Escherichia coli* is one of the most important bacteria that causes resistance problem. These bacteria produce an enzyme called extended-spectrum β-lactamase (ESBL) that allows it to become resistant to a wide variety of penicillins and cephalosporins. Currently, no information or published studies on ESBL-producing *E.coli* in broilers are available in the Philippines.

This cross-sectional study was conducted to determine the prevalence and distribution of extended-spectrum β-lactamase (ESBL)-encoding genes, *bla*_CTX-M_, *bla*_SHV_, and *bla*_TEM_*,* among *E. coli* isolates from broiler farms in Luzon, Philippines.

**Results:**

Results showed a farm prevalence of 66. 67%. A total of 69 (44.23%) ESBL-producing *E. coli* were isolated from boot swabs and cloacal swab samples from broiler farms. All major *bla*_CTX-M_ groups except *bla*_CTX-M-25_ group were identified in the isolates. The most prevalent group was *bla*_CTX-M-1_, 72.46% (CI: 60.38–82.54%), followed by *bla*_CTX-M-2_, *bla*_CTX-M-9_ group and *bla*_CTX-M-8_. The *bla*_TEM_ and *bla*_SHV_ genes were identified in 57.97 and 27.54% of isolates, respectively. The *bla*_CTX-M_ and *bla*_TEM_ were the most common gene combinations (33.33%). Coexistence of *bla*_CTX-M_ types was observed in 50 (73.53%) isolates.

**Conclusion:**

This study shows the high prevalence, diversity of patterns and coexistence of ESBL genes in the *E. coli* isolates from cloacal and boot swabs from broiler farms which pose risks of possible transmission to the environment, other animals and human.

## Background

Antimicrobial resistance (AMR) has become a rapidly growing public health concern worldwide. Infections from resistant bacteria are now too common, and some pathogens have even become resistant to multiple types of antibiotics. The Food and Agriculture Organization of the United Nations (FAO) estimates that around 500,000 human deaths related to antimicrobial resistance occur each year and AMR threat is believed to become more intense by 2050 leading to an estimated 10 million deaths annually [1].

One specific AMR problem with global spread affecting both animals and humans is the extended-spectrum beta-lactamase (ESBL)-producing *E. coli* [2]. These bacteria are resistant to penicillins, cephalosporins, and aztreonam mainly due to the production of CTX-M, TEM and SHV β-lactamases which are encoded by *bla*_CTX-M_, *bla*_SHV_, and *bla*_TEM_ genes, respectively. These genes can be plasmid-mediated or expressed chromosomally. Among these three, CTX-M-enzymes have become the most widespread type of ESBL in animals and humans. The name CTX reflects the potent hydrolytic activity of these β-lactamases against cefotaxime and they are not very closely related to TEM or SHV β-lactamases [3, 4].

The presence of ESBL-producing *E. coli* (ESBL-EC) in food animal production systems poses public health concern since it can be transmitted to humans via the food chain [5, 6]. Transmission of ESBL-EC in broiler farming was described previously wherein farm workers shared the same plasmid family and *E. coli* sequence type with broiler isolates [7]. Human infection due to ESBL-producing bacteria is associated with increased mortality, morbidity, high cost of hospitalization, and delay in appropriate therapy [2].

Currently, there is a lack of information on the occurrence of ESBL Enterobacteriaceae in broiler farms in the Philippines unlike the regular antimicrobial resistance surveillance program among humans in various hospitals in the country in the past decades [8–10]. The identification of the presence of ESBL genes in isolates from broiler farms will be useful in formulating evidence-based policies on mitigating antimicrobial resistance.

Hence, this study determined the prevalence and distribution of extended- spectrum β-lactamase-encoding genes, *bla*_CTX-M_, *bla*_SHV_, *bla*_TEM_ among ESBL-EC isolates from commercial broiler farms in Luzon, Philippines.

## Results

### Prevalence of ESBL *E. coli* in farms and samples

The prevalence of ESBL E.coli in the selected farms was 66. 67% (52/78). There is no significant difference in the farm prevalence in four provinces. A total of 69 (44.23%) ESBL-EC were isolated and these came from 47 pooled cloacal swab (60.26%) and 22 boot swab (28.21%) samples from broilers farms in Luzon, Philippines (Table 1). There is a significant difference in the prevalence between cloacal swab and boot swab samples (*p* < 0.05), with lower ESBL-EC isolates recovered from the latter.

**Table 1:** Prevalence of ESBL-producing *E. coli* in broiler farms (*n* = 78) in selected provinces in Luzon

Farm/Samples	No. of Positives	Prevalence %	95% Confidence Interval		*P* Value*
			Lower	Upper	
Farm	52	66.67	55.08	76.94	
Province 1	4	44.44	13.70	78.80	
Province 2	26	66.67	49.78	80.91	
Province 3	16	80.00	56.34	94.27	
Province 4	6	60.00	26.24	87.84	
Pooled Cloacal Swabs	47	60.26	48.54	71.17	< 0.0001
Boot Swabs	22	28.21	18.59	39.53	

* There is a significant difference in the prevalence between cloacal and boot swab samples

### Antimicrobial resistance profile of isolates

**Fig. 1:**
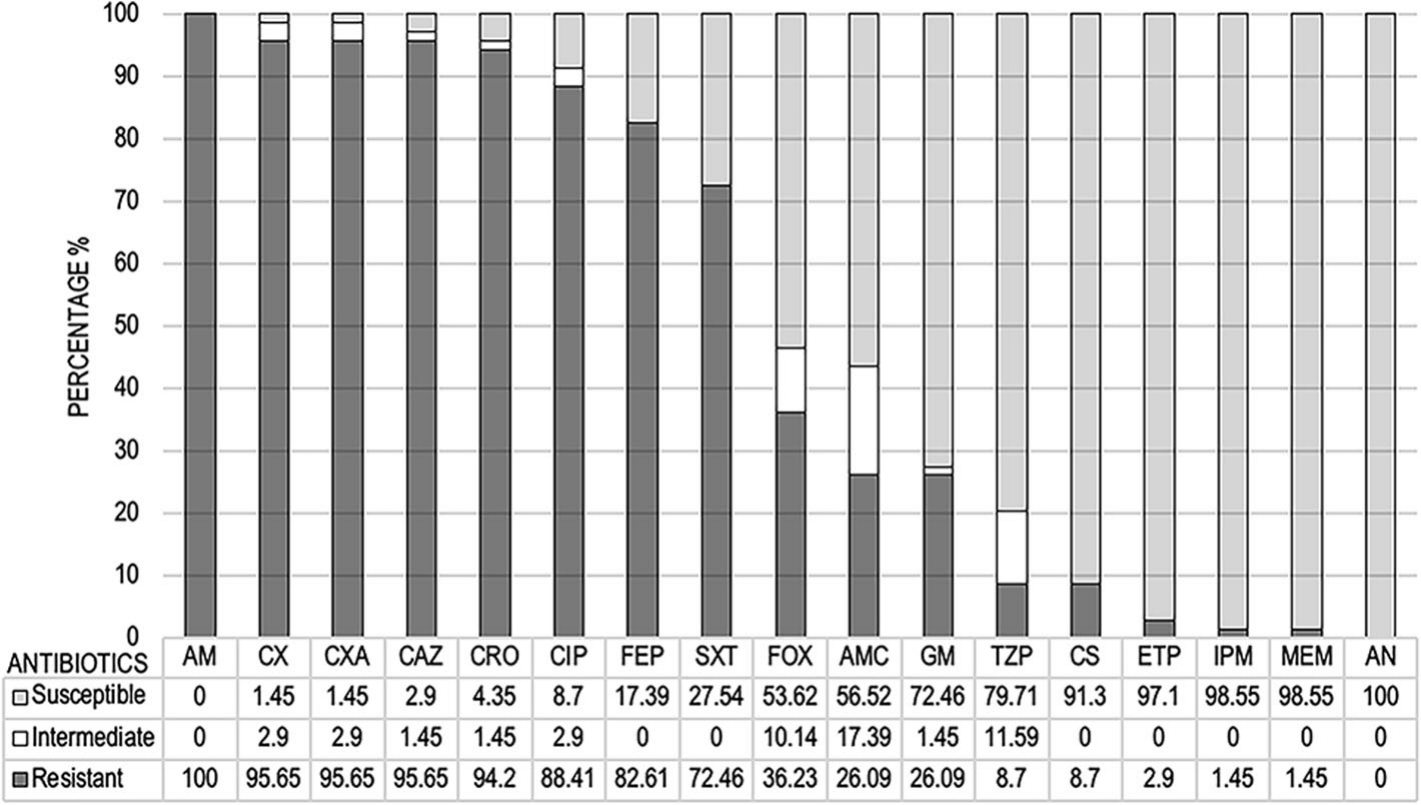
Antimicrobial resistance pattern of ESBL-producing *E. coli* isolates from broiler farms. Ampicillin (AM), amoxicillin/clavulanic acid (AMC), piperacillin/tazobactam (TZP), cefuroxime (CX), cefuroxime axetil (CXA), cefoxitin (FOX), ceftazidime (CAZ), ceftriaxone (CRO), cefepime (FEP), ertapenem (ETP) imipenem (IPM), meropenem (MEM), amikacin (AN), gentamicin (GM), ciprofloxacin (CIP), colistin (CS), trimethoprim/sulfamethoxazole (SXT)

Following the CLSI (M100-S24) interpretive criteria, the isolates showed phenotypic resistance to ampicillin (100%) and most cephems (92.75%) except cefoxitin (36.23%). Additionally, the isolates also showed very high resistance to ciprofloxacin (88.41%) and trimethoprim/sulfamethoxazole (72.46%). Resistance to colistin and carbapenem were detected in 8.70 and 2.90% of isolates, respectively. Figure 1 showed the antimicrobial resistance pattern of ESBL-EC isolates from broiler farms.

### Prevalence of ESBL genes

The most prevalent *bla*_CTX-M_ group among broiler isolates is *bla*_CTX-M-1_ group (72.46%), followed by *bla*_CTX-M-2_ group (65.22%) and *bla*_CTX-M-9_ group (52.17%). In addition to *bla*_CTX-M_ genes, *bla*_TEM_ and *bla*_SHV_ genes were also identified in 57.97 and 27.54% of poultry isolates, respectively. The prevalence of ESBL-EC resistance genes among cloacal and boot swab samples were summarized in Table 2.

**Table 2:** Prevalence and confidence interval of ESBL-producing *E. coli* resistance genes detected in cloacal and boot swabs from broiler farms (*n* = 69)

Resistance Genes	Cloacal swabs				Boot swabs				Total			
	*n*	Prev %	95% CI		*n*	Prev %	95% CI		*n*	Prev %	95% CI	
			LL	UL			LL	UL			LL	UL
*bla*_CTX-M_	40	57.97	45.48	69.76	22	31.88	21.17	44.21	62	89.86	80.21	95.82
*bla*_CTX-M-1_	35	50.72	38.41	62.98	15	21.74	12.71	33.31	50	72.46	60.38	82.54
*bla*_CTX-M-15_	35	50.72	38.41	62.98	15	21.74	12.71	33.31	50	72.46	60.38	82.54
*bla*_CTX-M-2_	31	44.93	32.92	57.38	14	20.29	11.56	31.69	45	65.22	52.79	76.29
*bla*_CTX-M-8_	10	14.49	7.17	25.04	5	7.25	2.39	16.11	15	21.74	12.71	33.31
*bla*_CTX-M-9_	22	31.88	21.17	44.21	14	20.29	11.56	31.69	36	52.17	39.80	64.35
*bla*_CTX-M-25_	0	–	–	–	0	–	–	–	0	–	–	–
*bla*_TEM_	29	42.03	30.24	54.52	11	15.94	8.24	26.74	40	57.97	45.48	69.76
*bla*_SHV_	14	20.29	11.56	31.69	5	7.25	2.39	16.11	19	27.54	17.46	39.62

### Distribution of ESBL genotypes

The distribution of main ESBL genotypes among isolates was presented in Table 3 while the distribution patterns of *bla*_CTX-M_ groups in the isolates were presented in Table 4. Coexistence of *bla*_CTX-M_ types was observed in 50 (73.53%) isolates while 12 (17.65%) and 6 (8.82%) isolates had only *bla*_CTX-M-1_ and *bla*_CTX-M-2_, respectively. A total of 9 isolates (13.04%) have genotypic resistance pattern combinations of *bla*_CTX-M-1_, *bla*_CTX-M-2_, *bla*_CTX-M-9_, *bla*_CTX-M-15_, and *bla*_TEM_ while 7 isolates (10.14%) have the same genotypic pattern, with the addition of *bla*_SHV_.

**Table 3:** Distribution of ESBL genotype among ESBL-producing *E. coli* isolates from broiler farms

Patterns of ESBL genotype	No. of isolates	Percentage
*bla*_CTX-M_ + *bla*_TEM_ + *bla*_SHV_	15	21.74
*bla*_CTX-M_ + *bla*_TEM_	23	33.33
*bla*_CTX-M_ + *bla*_SHV_	4	5.80
*bla*_CTX-M_ only	26	37.68
*bla*_TEM_ only	1	1.45
Total	69	100

**Table 4:** Distribution of *bla*_CTX-M_ groups in ESBL- producing *E. coli* isolates from broiler farms

Patterns of *bla*_CTX-M groups_	No. of isolates	Percentage
*bla*_CTX-M-1_ + *bla*_CTX-M-2_ + *bla*_CTX-M-9_	23	33.82
*bla*_CTX-M-1_ + *bla*_CTX-M-2_	14	20.59
*bla*_CTX-M-1_ + *bla*_CTX-M-9_	11	16.18
*bla*_CTX-M-2_ + *bla*_CTX-M-9_	2	2.94
*bla*_CTX-M-1_	12	17.65
*bla*_CTX-M-2_	6	8.82
Total	68	100

## Discussion

ESBL-producing *E. coli* (ESBL-EC) isolated from livestock and poultry animals is of public health concern since infections with these bacteria can result to treatment failure using commonly used penicillins and cephalosporins which increases the risk of mortality and delay in appropriate treatment [2]. Though ESBL-EC can be susceptible to certain cephalosporins and penicillins/β-lactamase inhibitors combinations, these drugs are rarely used as first line of treatment in *E. coli* infections.

This is the first report of ESBL-EC in broiler farms in the Philippines with the very high prevalence as well as phenotypic and genotypic resistance patterns. A farm prevalence of 66.67% (52/78) is alarming and requires risk assessments and appropriate risk management to minimize the occurrence and spread of this resistant pathogen. With *E. coli* as a major opportunistic pathogen in broiler chickens and with a potential for zoonotic transfer to human, ESBL-EC represents a major risk both to poultry production and to human health [11]. Seventeen farms have positive isolates both from cloacal and boot swab samples. The rest of the farms were either positive for cloacal swabs or boot swabs.

The most prevalent ESBL encoding gene in this study is *bla*_CTX-M_ which is similar to published studies in poultry [12–14]. In humans, however, the recent report revealed that TEM-type is more prevalent in clinical isolates from Filipinos [8] which is contrary to earlier reports wherein *bla*_CTX-M_ as the most prevalent type in hospitalized patients [15]. Previous studies suggest that ESBL genotypes can vary between regions and geographical location. Therefore, it is warranted to conduct wider scope and regular surveillance study to determine the prevalence and distribution of these enzymes among broiler farms in the Philippines.

We identified *bla*_CTX-M-1_ and *bla*_CTX-M-15_ genes as the most prevalent *bla*_CTX-M_ variants in this study which is similar to other reports on poultry [14] and humans [16]. Some studies also established the relationship of poultry isolates from human isolates suggesting a potential zoonotic transmission [7]. This could be the result of faecal contamination of poultry meat during slaughter, processing, selling and cooking of poultry products [5, 17]. Moreover, the high prevalence of *bla*_CTX-M-15_ gene in this study has public health concern since it is the most widespread gene type of ESBL-EC in humans [18].

The detection of ESBL-EC in boot swabs in this study suggests the possible spread of the pathogen in the environment which could be a factor for a transmission in farm workers and in the community as previously reported [7, 13]. In this study, a significantly lower prevalence of ESBL-EC were isolated from boot swabs compared to cloacal swabs (*p* < 0.05). This can be expected especially when the farms have good management practices and the floorings are kept dry [11]. Despite the lower number of ESBL-EC isolates in boot swabs, our result shows *bla*_CTX-M-15_ as the most prevalent (21.74%) genotype in boot swab samples similar to cloacal swab samples suggesting horizontal transmission to the environment. However, we were not able to establish which came first as the previous study showed that ESBL-EC -free day-old chicks can acquire the pathogen upon entry at the farm [19]. To reduce the risk of transmission, ESBL-EC should be either eliminated from poultry production or reduced the occurrence to levels with lower risk of spread to humans [11].

The *bla*_CTX-M-2_ was the third most common *bla*_CTX-M_ type but the second most common group (since both *bla*_CTX-M-1_ and *bla*_CTX-M-15_ belong to the *bla*_CTX-M-1_ group) in this study. It was previously isolated in chicken meat and in healthy chickens [17, 19]. The *bla*_CTX-M-9_ gene was observed in 52.17% of isolates in this study. The *bla*_CTX-M-9_ gene is widely reported in earlier studies in human infections in Europe, particularly in Spain and UK. A study in 2003 also reported the occurrence of these genes in poultry isolates in France. The CTX-M-9-like enzymes (CTX-M9 and CTX-M-14) have been linked directly or indirectly with animals in different countries [20].

Most of the isolates from poultry carry two or more *bla*_CTX-M_ groups. A total of 23 (33.82%) poultry isolates have three types of *bla*_CTX-M_. In this study, co-existence of two or more CTX-M-type β-lactamases in the same strain is common. This coexistence of different types of CTX-M can be a normal scenario since they have many homologous regions which may result in the emergence of recombinant enzymes [18, 21]. We speculate that multiple CTX-M types in single isolate could imply that infections caused by these isolates may be more difficult to treat since ESBL expression is more likely to occur phenotypically.

The coexistence of different β-lactamase genes within the same isolates has been reported by several investigators [14, 21]. The most common ESBL genotype among our isolates was *bla*_CTX-M_ and *bla*_TEM_ (33.33%) which agrees with other studies [22]. The *bla*_CTX-M_ gene with the *bla*_TEM_ gene is the most common combination with or without *bla*_SHV_ in this study which corroborates with the previous report detecting these three genotypes in poultry cloacal swab samples [23]. To our knowledge, this is the first report of high co-resistance pattern among poultry isolates in the Philippines. The presence of multiple ESBL resistance genes could result in retained resistance to β-lactamases despite the reduced expression of one or two genes.

Antimicrobial susceptibility testing showed 100% resistance to ampicillin. Studies have shown that *bla*_TEM_ gene is highly prevalent in samples of chickens and human with ampicillin resistant-*E. coli*. [24] Colistin resistance was observed in six isolates. Colistin is considered as a last resort antibiotic for treating multi-drug resistant Enterobacteriaceae. Detection of *mcr*, the gene responsible for colistin resistance, in ESBL-EC from poultry samples would augment the public health importance of monitoring the antimicrobial usage in poultry farms. Likewise, very high resistance to ciprofloxacin (88.41%) was observed and this points to the possibility of ST131 circulating at high prevalence in the flocks which should be further studied. We also detected carbapenem resistance (2.90%) in our isolates. These findings warrant further investigation of the presence of carbapenem resistance genes since such resistant pathogens are among the list of World Health Organization (WHO) top priority pathogens for the development of antimicrobials. We suggest detecting the presence of a plasmid-mediated *bla*_NDM-1_ gene encoding the metallo-β-lactamase NDM-1 which hydrolyze beta-lactam antibiotics including carbapenems. Moreover, plasmids encoding for ESBL can be transferred from the *E. coli* poultry strains to human while carrying other antibiotic and resistance genes [25]. Some controversies are arising whether antimicrobial usage is the main contributing factor in the positivity of some broiler farms since study have shown the occurrence of ESBL-EC in farms with no or limited use of antibiotics [19, 26].

Although we have not yet subjected all the PCR products for DNA sequencing, we believe that the PCR amplification of *bla*_CTX-M_-specific products alone and without sequencing usually provides sufficient evidence that a *bla*_CTX-M_ gene is responsible for the expressed phenotype. However, further analysis should be conducted in *bla*_TEM_ and *bla*_SHV_ since sequencing is essential to discriminate between the non-ESBL parent enzymes (TEM1, TEM2, or SHV1) and different variants of TEM or SHV ESBLs (TEM3, SHV2) [27]. In addition, multilocus sequence typing and whole genome sequencing should be performed to further elucidate the chromosomal backgrounds of strains harboring these genes.

We believe that ESBL-EC at low bacterial population in the samples may have not been isolated and identified thus alternatively, we suggest that direct PCR-based detection can be employed. The universal CTX-M primer was not able to detect all positive samples (89.86%) despite showing positive results to other CTX-M group primers. In addition, there were also nine *bla*_CTX-M_-_15_ samples but were negative to the *bla*_CTX-M-1_ primer. We suggest the use and development of multiplex PCR to minimize such problems. Further molecular analyses could be performed to establish the relatedness of the ESBL-EC from the broiler samples to human isolates since the antimicrobial resistance genes evaluated in this study can be easily transferred to animal and human strains. In addition, further study on the isolates should be conducted to describe the connection between the presence and degree of expression of the selected genes.

## Conclusions

In conclusion, results reveal the occurrence of the three major ESBL genotypes, *bla*_CTX-M_, *bla*_TEM_, and *bla*_SHV_, and the major groupings of CTX-M enzymes in *E. coli* isolates from cloacal and boot swab samples from broiler farms. The high prevalence, diversity of patterns and coexistence of these genotypes in the bacterial isolates is alarming. Further surveillance study in the Philippines is necessary to document the rapid emergence and spread of multi-resistant ESBL-EC in broiler production system and the food chain.

## Materials and methods

### Farm selection

**Fig. 2:**
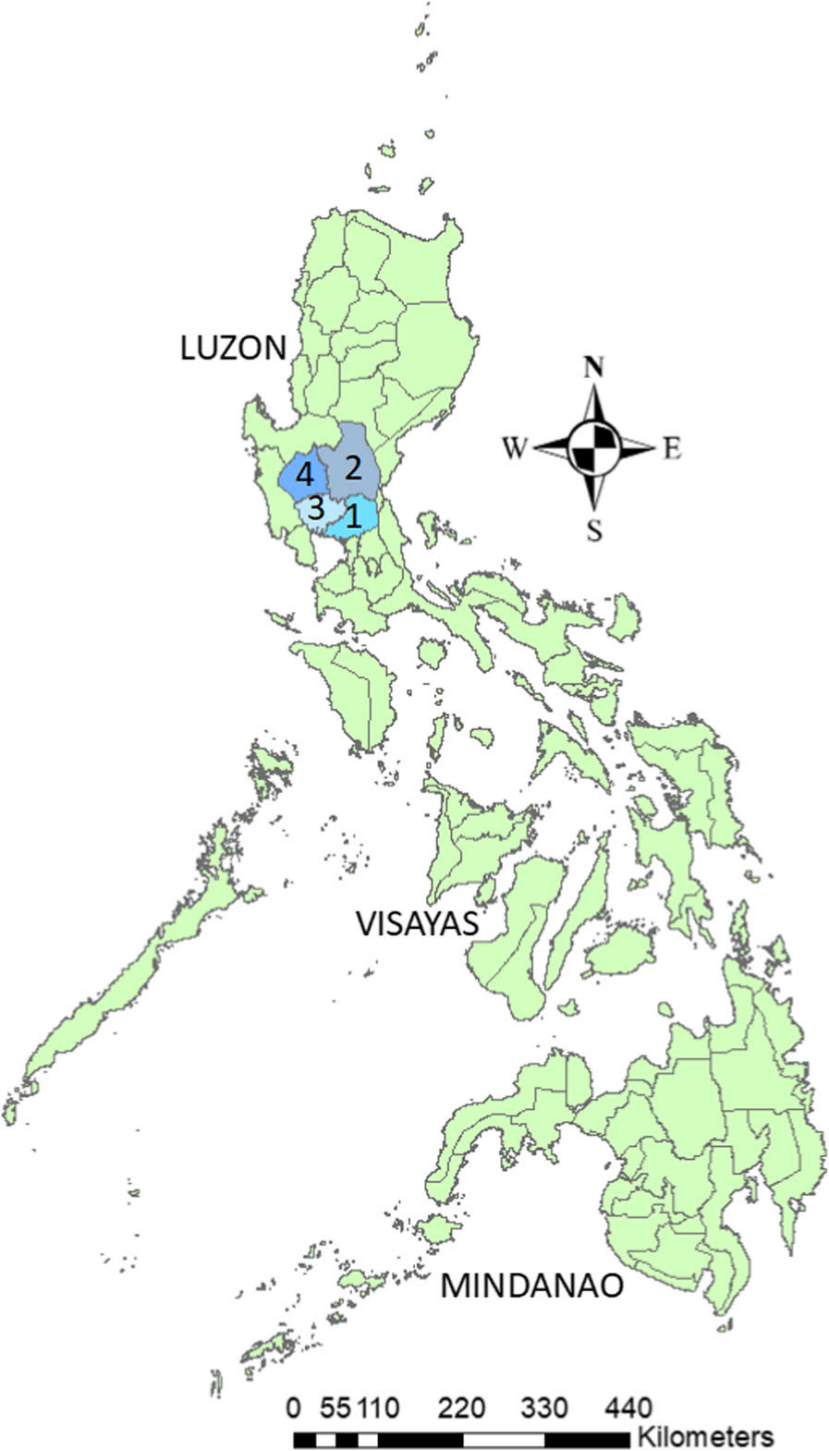
Map of the Philippines showing the study provinces. Map originally prepared by the authors and no copyright permission is required

The four provinces in Luzon (Fig. 2) with the highest broiler production in the central region were selected. From these provinces, a sampling frame of all broiler farms were constructed using the information on the number of existing farms obtained from the Provincial Veterinary Offices of each province. A total of 391 broiler farms were identified from four study provinces and the sample size was calculated using the following assumptions: 50% prevalence, 10% accepted error and 95% level of confidence. Using probability proportional to size sampling, a total of 78 sample farms were randomly selected from Province 1 (9 out of 44), Province 2 (39 out of 197), Province 3 (20 out of 101) and Province 4 (10 out of 49). Out of 78 selected farms, 28 operate commercially while the other 50 are in contract growing operation under five companies. These farms have a mean broiler population of 68,872 birds. Each selected farm was contacted for the collection of samples and sampling was performed during the months of March to June, 2017.

### Sampling and bacterial isolation

For each selected broiler farm, cloacal swabs were collected using sterile cotton swabs directly from cloaca of 10 randomly selected birds. The cloacal swab samples in each farm were pooled in Falcon tubes containing 25 ml Luria-Bertani (LB) broth (Merck, Darmstadt, Germany). A paired boot swab samples were obtained by walking along the whole length of the broiler house. Boot swab samples were placed in a 500 ml beaker containing 250 ml of LB broth for enrichment. A total of 156 samples (78 pooled cloacal swabs and 78 boot swabs) from 78 broiler farms were processed and subjected to microbiological analysis. Samples were incubated aerobically at 37 °C for 18–24 h. Thereafter, a loopful (10 μl) of each enriched sample was streaked onto MacConkey agar plate (Oxoid, United Kingdom) supplemented with 1 mg/L cefotaxime and incubated aerobically at 37 °C for 24 h. A replicate MacConkey agar plate without cefotaxime was also prepared for each sample. Subsequently, one bright pink colony, suggestive of lactose-fermenting bacteria and morphologically indicative of *E. coli,* was picked and streaked in a selective and differential medium, Eosin Methylene Blue agar plate (HiMedia, Mumbai, India) and incubated at 37 °C for 24 h. The bacteria isolated from all pooled fecal and swab samples were identified.

### Bacterial identification and antimicrobial susceptibility testing

Bacterial identification and antimicrobial susceptibility tests were performed through Vitek® 2 Compact (bioMérieux, Craponne, France), an automated microbiology system utilizing growth-based technology, using GN and AST-N261 cards, respectively. Combined disc method was also done on all presumptive ESBL-EC isolates to confirm ESBL production. Both ceftazidime (30 μg) and cefotaxime (30 μg) alone and in combination with 10 μg clavulanic acid were tested. A ≥ 5 mm increase in the zone diameter for either antimicrobial agent tested in combination with clavulanic acid versus its zone when tested alone confirmed the presence of an ESBL [28]. For quality control, *E. coli* ATCC 25922 (Microbiologics, Minnesota, USA) was used in both Vitek® 2 Compact and Combined disc method for the screening and confirmatory testing of ESBL-producing *E. coli* as well as antimicrobial susceptibility testing.

### DNA extraction

For DNA extraction of bacterial isolates, the column isolation method using NucleoSpin Microbial DNA (Macherey-Nagel, Germany) was performed following manufacturer’s protocol.

### PCR amplification

PCR amplifications were carried out using the optimized conditions from published studies (Table 5). All isolates were screened for target genes. The PCR assay was performed in BioRad T100 thermal cycler (BioRad, Herts, United Kingdom) individually for each primer set according to the following amplification conditions: initial denaturation at 95 °C for 3 min, 35 cycles of denaturation at 94 °C for 1 min, and optimized annealing temperature for each primer set (Table 5). Elongation was set at 72 °C for 1 min with final elongation at 72 °C for 7 min. One microliter of *E.coli* DNA lysate was used as template for the PCR reaction mixture containing 0.5 U DNA taq polymerase, 1x PCR buffer, 2 Mm MgCl_2_, 1 mM dNTP, 1 uM each of primer pair. A mixture of 3 μl of PCR products and 2 μl of loading buffer was loaded in 1.5% agarose gel and separated through electrophoresis using 0.5x TBE buffer to determine the molecular size of the amplified products per target gene. *E. coli* strains of ATCC 25922 and ATCC 35218 (β-lactamase-producing strain) (Microbiologics, Minnesota, USA) were used as negative and positive controls in the PCR, respectively. Purified PCR products from few representative isolates were sent to 1st Base Laboratories (Axil Scientific Pte Ltd., Singapore) for DNA sequencing analysis to confirm the target genes. Matches were analysed using Basic Local Alignment Search Tool (BLAST).

**Table 5:** Primers used to detect ESBL-resistance genes and genotypes in broiler farm isolates

Target gene	Primer	Sequence (5 → 3)	Annealing Temp (°C)	Size (bp)	Ref
*bla*_CTX-M_	CTX-M-F CTX-M-R	ATGTGCAGYACCAGTAARGTKATGGC TGGGTRAARTARGTSACCAGAAYSAGCGG	55	592	[29]
*bla*_CTX-M-1group_	CTX-M-1-F CTX-M-1-R	GGTTAAAAAATCACTGCGTC TTACAAACCGTYGGTGACGA	50	873	[29]
*bla*_CTX-M-15_	CTX-M-15-F CTX-M-15-R	CACACGTGGAATTTAGGGACT GCCGTCTAAGGCGATAAACA	50	995	[30]
*bla*_CTX-M-2group_	CTX-M-2-F CTX-M-2-R	ATGATGACTCAGAGCATTCGCCGC TCAGAAACCGTGGGTTACGATTTT	56	876	[31]
*bla*_CTX-M-8group_	CTX-M-8-F CTX-M-8-R	TGATGAGACATCGCGTTAAG TAACCGTCGGTGACGATTTT	52	666	[32]
*bla*_CTX-M-9group_	CTX-M-9-F CTX-M-9-R	GTGACAAAGAGAGTGCAACGG ATGATTCTCGCCGCTGAAGCC	55	856	[33]
*bla*_CTX-M-25group_	CTX-M-25-F CTX-M-25-R	GCACGATGACATTCGGG AACCCACGATGTGGGTAGC	52	327	[34]
*bla*_TEM_	TEM-F TEM-R	TTGGGTGCACGAGTGGGTTA TAATTGTTGCCGGGAAGCTA	55	506	[35]
*bla*_SHV_	SHV-F SHV-R	TCGGGCCGCGTAGGCATGAT AGCAGGGCGACAATCCCGCG	52	628	[35]

### Statistical analysis

The data were analyzed descriptively. Farm prevalence was calculated as the number of farms with at least one positive isolate, either from cloacal swabs or boot swabs, over the total number of farms studied. The 95% confidence intervals were determined using exact binomial confidence limits for the proportion with a significance level (alpha) of 0.05, to test for the difference in proportions.

## Data Availability

All data generated or analysed in this study are included in this published article. The detailed raw data are available from the corresponding author on reasonable request.

## Abbreviations

AM: Ampicillin
AMC: Amoxicillin/Clavulanic Acid
AMR: Antimicrobial Resistance
AN: Amikacin
AST: Antimicrobial Susceptibility Test
CAZ: Ceftazidime
CDT: Combined Disc Test
CIP: Ciprofloxacin
CLSI: Clinical and Laboratory Standards Institute
CRO: Ceftriaxone
CS: Colistin
CXM: Cefuroxime
CXMA: Cefuroxime Axetil
EMB: Eosin Methylene Blue Agar
ESBL-EC: Extended Spectrum Beta-Lactamase producing *E. coli*
ETP: Ertapenem
FEP: Cefepime
FOX: Cefoxitin
GM: Gentamicin
IPM: Imipenem
MAC: MacConkey Agar
MEM: Meropenem
PCR: Polymerase Chain Reaction
SXT: Trimethoprim/Sulfamethoxazole
TZP: Piperacillin/Tazobactam

## References

[1] Reardon Sara (2014). Phage therapy gets revitalized. Nature.

[2] Chong Y, Shimoda S, Shimono N (2018). Current epidemiology, genetic evolution and clinical impact of extended-spectrum β-lactamase-producing *Escherichia coli* and *Klebsiella pneumoniae*. Infect Genet Evol.

[3] Michael GB, Freitag C, Wendlandt S, Eidam C, Feßler AT, Lopes GV (2015). Emerging issues in antimicrobial resistance of bacteria from food-producing animals. Future Microbiol.

[4] Shaikh S, Fatima J, Shakil S, Rizvi SMD, Kamal MA (2015). Antibiotic resistance and extended spectrum beta-lactamases: types, epidemiology and treatment. Saudi J Biol Sci.

[5] Boonyasiri A, Tangkoskul T, Seenama C, Saiyarin J, Tiengrim S, Thamlikitkul V (2014). Prevalence of antibiotic resistant bacteria in healthy adults, foods, food animals, and the environment in selected areas in Thailand. Pathog Glob Health.

[6] Tekiner I, Ozpinar H (2016). Occurrence and characteristics of extended spectrum beta-lactamases-producing Enterobacteriaceae from foods of animal origin. Brazilian J Microbiol.

[7] Huijbers P. M. C., Graat E. A. M., Haenen A. P. J., van Santen M. G., van Essen-Zandbergen A., Mevius D. J., van Duijkeren E., van Hoek A. H. A. M. (2014). Extended-spectrum and AmpC β-lactamase-producing Escherichia coli in broilers and people living and/or working on broiler farms: prevalence, risk factors and molecular characteristics. Journal of Antimicrobial Chemotherapy.

[8] Cruz MC, Hedreyda CT (2017). Detection of plasmid-borne β-lactamase genes in extended-spectrum β-lactamase (ESBL) and non-ESBL-producing *Escherichia coli* clinical isolates. Philipp J Sci.

[9] Cabrera EC, Rodriguez RD (2009). First report on the occurrence of SHV-12 extended-spectrum beta-lactamase-producing Enterobacteriaceae in the Philippines. J Microbiol Immunol Infect.

[10] Kanamori H, Navarro RB, Yano H, Sombrero LT, Capeding MRZ, Lupisan SP (2011). Molecular characteristics of extended-spectrum β-lactamases in clinical isolates of Enterobacteriaceae from the Philippines. Acta Trop.

[11] Olsen RH, Bisgaard M, Löhren U, Robineau B, Christensen H (2014). Extended-spectrum β-lactamase-producing *Escherichia coli* isolated from poultry: a review of current problems, illustrated with some laboratory findings. Avian Pathol.

[12] Shin SW, Jung M, Won HG, Belaynehe KM, Yoon IJ, Yoo HS (2017). Characteristics of transmissible CTX-M- and CMY-type β -lactamase- producing *Escherichia coli* isolates collected from pig and chicken farms in South Korea. J Microbiol Biotechonology.

[13] Bui TKN, Bui TMH, Ueda S, Le DT, Yamamoto Y, Hirai I (2018). Potential transmission opportunity of CTX-M-producing *Escherichia coli* on a large-scale chicken farm in Vietnam. J Glob Antimicrob Resist.

[14] Li S, Zhao M, Liu J, Zhou Y, Miao Z (2016). Prevalence and antibiotic resistance profiles of extended-Spectrum β-lactamase–producing *Escherichia coli* isolated from healthy broilers in Shandong Province, China. J Food Prot.

[15] Tian GB, Garcia J, Adams-Haduch JM, Evangelista JP, Destura RV, Wang HN (2010). CTX-M as the predominant extended-spectrum β-lactamases among Enterobacteriaceae in Manila, Philippines. J Antimicrob Chemother.

[16] Maciuca IE, Williams NJ, Tuchilus C, Dorneanu O, Guguianu E, Carp-Carare C (2015). High prevalence of *Escherichia coli-* producing CTX-M-15 extended-Spectrum Beta-lactamases in poultry and human clinical isolates in Romania. Microb Drug Resist.

[17] Aliyu AB, Saleha AA, Jalila A, Zunita Z (2016). Risk factors and spatial distribution of extended spectrum β-lactamase-producing- *Escherichia coli* at retail poultry meat markets in Malaysia: a cross-sectional study. BMC Public Health.

[18] Cantón R, González-Alba JM, Galán JC (2012). CTX-M enzymes: origin and diffusion. Front Microbiol.

[19] Huijbers PMC, Graat EAM, van Hoek AHAM, Veenman C, de Jong MCM, van Duijkeren E (2016). Transmission dynamics of extended-spectrum β-lactamase and AmpC β-lactamase-producing *Escherichia coli* in a broiler flock without antibiotic use. Prev Vet Med.

[20] Coque TM, Baquero F, Canton R (2008). Increasing prevalence of ESBL-producing Enterbacteriaceae in Europe. Eurosurveillance.

[21] He D, Partridge SR, Shen J, Zeng Z, Liu L, Rao L (2013). CTX-M-123, a novel hybrid of the CTX-M-1 and CTX-M-9 group β-lactamases recovered from *Escherichia coli* isolates in China. Antimicrob Agents Chemother.

[22] Khoshbakht R, Seifi S, Raeisi M (2016). Antibiotic susceptibility and high prevalence of extended spectrum beta-lactamase producing *Escherichia coli* in iranian broilers. Rev Med Vet (Toulouse).

[23] Selma C, Hamza L, Bernard D, Atef A, Rolain J-M (2017). Prevalence of extended-spectrum beta lactamase and carbapenemase-encoding genes in poultry feces from Algeria and Marseille, France. J Glob Antimicrob Resist.

[24] Hemeg HA (2018). Molecular characterization of antibiotic resistant *Escherichia coli* isolates recovered from food samples and outpatient clinics, KSA. Saudi J Biol Sci.

[25] Wang J, Stephan R, Karczmarczyk M, Yan Q, Hächler H, Fanning S (2013). Molecular characterization of blaESBL-harboring conjugative plasmids identified in multi-drug resistant *Escherichia coli* isolated from food-producing animals and healthy humans. Front Microbiol.

[26] Casella T, Nogueira MCL, Saras E, Haenni M, Madec JY (2017). High prevalence of ESBLs in retail chicken meat despite reduced use of antimicrobials in chicken production, France. Int J Food Microbiol.

[27] Pitout JD, Laupland KB (2008). Extended-spectrum β-lactamase-producing Enterobacteriaceae: an emerging public-health concern. Lancet Infect Dis.

[28] Kalil Andre C., Metersky Mark L., Klompas Michael, Muscedere John, Sweeney Daniel A., Palmer Lucy B., Napolitano Lena M., O'Grady Naomi P., Bartlett John G., Carratalà Jordi, El Solh Ali A., Ewig Santiago, Fey Paul D., File Thomas M., Restrepo Marcos I., Roberts Jason A., Waterer Grant W., Cruse Peggy, Knight Shandra L., Brozek Jan L. (2016). Management of Adults With Hospital-acquired and Ventilator-associated Pneumonia: 2016 Clinical Practice Guidelines by the Infectious Diseases Society of America and the American Thoracic Society. Clinical Infectious Diseases.

[29] Moubareck C, Daoud Z, Hakimé NI, Hamzé M, Mangeney N, Matta H (2005). Countrywide spread of community- and hospital-acquired extended-spectrum β-lactamase (CTX-M-15)-producing Enterobacteriaceae in Lebanon. J Clin Microbiol.

[30] Muzaheed YD, Adams-Haduch JM, Endimiani A, Sidjabat HE, Gaddad SM, Paterson DL (2008). High prevalence of CTX-M-15-producing Klebsiella pneumoniae among inpatients and outpatients with urinary tract infection in southern India. J Antimicrob Chemother.

[31] Celenza G, Pellegrini C, Caccamo M, Segatore B, Amicosante G, Perilli M (2006). Spread of *bla*_CTX-M_-type and blaPER-2 β-lactamase genes in clinical isolates from Bolivian hospitals. J Antimicrob Chemother.

[32] Jouini A, Vinué L, Ben SK, Sáenz Y, Klibi N, Hammami S (2007). Characterization of CTX-M and SHV extended-spectrum β-lactamases and associated resistance genes in *Escherichia coli* strains of food samples in Tunisia. J Antimicrob Chemother.

[33] Sabaté M, Tarragó R, Navarro F, Miró E, Vergés C, Barbé J (2000). Cloning and sequence of the gene encoding a novel cefotaxime-hydrolyzing β-lactamase (CTX-M-9) from *Escherichia coli* in Spain. Antimicrob Agents Chemother.

[34] Woodford N, Fagan EJ, Ellington MJ (2005). Multiplex PCR for rapid detection of genes encoding CTX-M extended-spectrum b -lactamases development of highly ciprofloxacin-resistant laboratory mutants of Acinetobacter baumannii lacking topoisomerase IV gene mutations. J Antimicrob Chemother.

[35] Melano R (2003). Multiple antibiotic-resistance mechanisms including a novel combination of extended-spectrum β-lactamases in a Klebsiella pneumoniae clinical strain isolated in Argentina. J Antimicrob Chemother.

